# Exogenous H_2_S modulates mitochondrial fusion–fission to inhibit vascular smooth muscle cell proliferation in a hyperglycemic state

**DOI:** 10.1186/s13578-016-0102-x

**Published:** 2016-05-31

**Authors:** Aili Sun, Yan Wang, Jiaqi Liu, Xiangjing Yu, Yu Sun, Fan Yang, Shiyun Dong, Jichao Wu, Yajun Zhao, Changqing Xu, Fanghao Lu, Weihua Zhang

**Affiliations:** Department of Pathophysiology, Harbin Medical University, Harbin, 150086 China; Department of Urologic Surgery, First Clinical Medical School of Harbin Medical University, Harbin, 150001 China

**Keywords:** Diabetes mellitus, Vascular smooth muscle cell, Hydrogen sulfide (H_2_S), Proliferation

## Abstract

**Aim:**

Vascular smooth muscle cell (VSMC) proliferation in response to hyperglycemia is an important process in the development of arterial vessel hyperplasia. The shape change of mitochondria is dynamic and closely related to fission and fusion. Hydrogen sulfide (H_2_S) was confirmed to have anti-oxidative, anti-inflammatory and anti-proliferative effects. However, little it is known about its effects on mitochondrial morphology induced by hyperglycemia. The aim of the study is to demonstrate that H_2_S inhibits VSMC proliferation through regulating mitochondrial fission.

**Methods and results:**

We observe lower H_2_S levels as well as higher proliferative protein expression levels for proliferative cell nuclear antigen (PCNA) and cyclin D1 and higher mitochondrial fusion–fission protein expression levels for dynamin-related protein 1 (Drp 1) in human kidney arteries and in db/db mouse aorta. Exogenous H_2_S (100 μM NaHS) inhibits vascular smooth muscle cells of human pulmonary aorta(HPASMC) proliferation and migration in response to high glucose using the BrdU and scratch wound repair assays, decreases proliferative protein (PCNA and cyclin D1) expression, and reduces ROS production in the cytoplasm and mitochondria. When HPASMCs proliferate with a high glucose treatment, the mitochondria become small spheres with a short rod-shaped structure, whereas NaHS, a mitochondrial division inhibitor and siDrp prevent VSMC proliferation and maintain mitochondria as stationary and randomly dispersed with fixed structures.

**Conclusion:**

Exogenous H_2_S aids in inhibiting mitochondrial fragmentation and affects proliferation in db/db mice and HPASMCs by decreasing Drp 1 expression.

**Electronic supplementary material:**

The online version of this article (doi:10.1186/s13578-016-0102-x) contains supplementary material, which is available to authorized users.

## Background

Diabetes mellitus is one of the most dangerous factors for cardiovascular disease. Diabetes cardiovascular complications account for 70 % of deaths in type 2 diabetic patients [1, 2]. Diabetes, atherosclerosis and post-percutaneous coronary intervention induce VSMC injury; VSMCs lose contractile function and convert to a high-proliferation phenotype [3]. Mounting evidence shows that chronic hyperglycemia increases reactive oxygen species (ROS) production and stimulates VSMC proliferation and migration, which, in aggregate, facilitates the development and progression of vascular pathology [4–7].

Mitochondria control virtually every aspect of cell function via changing the ATP concentration, contributing to Ca^2+^ signaling, influencing redox potential, and controlling ROS levels [8, 9]. Mitochondria are dynamic organelles that frequently move, undergo fission and fuse with other mitochondria to maintain their structure and function. Mitochondrial fission–fusion can aid in repairing defective mitochondria and plays a role in proper segregation of mitochondria into daughter cells during cell division [10]. Recent studies have demonstrated that, when cells enter a proliferative state, mitochondria become highly dynamic structures. When mitochondrial dynamics are inhibited, VSMCs proliferation is inhibited [11].

An important gasotransmitter in the cardiovascular system, H_2_S induces vascular relaxation and aids in regulating vascular smooth muscle cell proliferation [12, 13]. H_2_S can be generated in blood vessels in a reaction catalysed mainly by cystathionine gamma-lyase (CSE). Recent studies show that an abnormal metabolism and CSE/H_2_S pathway functions are linked to various cardiovascular diseases, including atherosclerosis and hypertension [14]. Yang et al. showed that SMCs-CSE Knock Out cells display a greater proliferation rate in vitro as well as in vivo, and the cells are more susceptible to apoptosis induced by exogenous H_2_S at physiologically relevant concentrations [15]. However, little known about the SMC proliferation mechanisms that decrease H_2_S production.

The purpose of the present study is to clarify whether the effect of ROS on mitochondrial dynamics as well as mitochondria fission and fusion are involved in smooth muscle cell proliferation in hyperglycemia and high glucose treatment. We also propose that H_2_S-mediated inhibition of SMC proliferation is related to mitochondria dynamics regulation.

## Methods

### Materials

Sodium hydrogen sulfide (NaHS),* N*-acetyl-cysteine (NAC, an inhibitor of reactive oxygen species, ROS), Mdivi-1 and palmitate were purchased from Sigma Chemical Co. (St. Lowis, MO, USA). Cyclin D1, p27, PCNA, p21 antibodies were obtained from Cell Signaling Technology (Danvers, USA). CSE, Drp1, Mfn2, MMP2, MMP9, Collagen I, and Collagen III were from ProteinTech Bioengineering Institute (Wuhan, China). Mitosox and Mitotracker green were purchased from Roche (Mannheim, Germany). All others chemicals were from Sigma or Santa Cruz.

### Patient recruitment and sample collection

The patients studied herein included 5 type 2 diabetes individuals with severe hydronephrosis and 4 individuals with severe hydronephrosis at the first affiliated hospital of Harbin Medical University, Harbin, P.R. China. Human renal artery tissues were obtained from these patients. Seventy percent of the subjects were male. This research ethics approval is from Harbin Medical University’s Research Ethics Board.

### Diabetic animal models

Ten-week-old female db/db mice and C57BL/6 mice were provided by the Animal Laboratory Center of Nanjing university. The genetically diabetic mouse (db/db) has a mutation on the chromosome 4 that inhibits the expression of leptin receptor. The syndrome of type 2 diabetes mellitus in db/db mice is similar to adult humans and is characterized by hyperinsulinemia, obesity and progressive hyperglycemia. The mice were maintained on a standard diet and water ad libitum. The experiments conformed to standard environmental conditions for temperature (20–22 °C) and humidity (50–60 %). Half of the db/db mice were treated with NaHS (100 µmol/Kg) for 8 weeks. The animal experimental protocols complied with the ‘Guide for the Care and Use of Laboratory Animals’ published by the United States National Institutes of Health. The study was approved by the Institutional Animal Research Committee of Harbin Medical University (Harbin, People’s Republic of China).

### Measurement of H_2_S concentrations and H_2_S production rate

H_2_S concentrations of the arteries were measured as described previously [16]. A total volume of 200 μl of artery homogenates was transferred directly into a tube containing zine acetate (1 % wt/vol, 187.5 μl) and NaOH (10 %, 12.5 μl) to trap the H_2_S for 15 min at room temperature without addition of exogenous CSE substrates or effectors. The reaction was terminated by adding 1 ml H_2_O, 200 μl of* N*,*N*-dimethyl-*P*-phenylenediamine sulfate (20 μM in 7.2 M HCl) and 200 μl of Feels(30 μM in 1.2 M HCl). After being kept in the dark for 15 min, 700 μl of mixture was added to a tube with 150 μl of trichloracetic acid (10 % wt/vol) to precipitate protein. Then the mixture was centrifuged at 10,000×*g* for 5 min and absorbance at 670 nm of the resulting supernatant (150 μl) was determined using a 96-well microplate reader. The H_2_S concentration of each sample was calculated against a calibration curve of NaHS.

The H_2_S production rate of arteries was described previously. The artery homogenates were sonicated in 50 mM ice-cold potassium phosphate buffer (pH 6.8). The flasks containing the reaction mixture (100 mM potassium phosphate buffer, 10 mM l-cysteine, 2 mM pyridoxal 5-phosphate, and 10 % wt/vol cell homogenates) and center wheels containing trapping solution of 0.5 ml 1 % zinc acetate and a piece of filter paper were flushed with N_2_ and incubated at 37 °C for 90 min. The reaction was stopped by adding 0.5 ml of 50 % trichloroacetic acid. The contents of the center wheels were transferred to test tubes, each containing 3.5 ml of water into which 0.5 ml of 20 μM* N*,*N*-dimetly-*P*-phenylenediamine sulfate in 7.2 M HCl and 0.5 ml of 30 mM FeCl_3_ were added. The absorbance of the resulting solution at 670 nm was measured 20 min later with a spectrophotometer.

### Vascular smooth muscle cells of human pulmonary aorta (HPASMC) culture

Vascular smooth muscle cells of human pulmonary aorta were maintained in DMEM containing 10 % fetal bovine serum (FBS) (Gibco-BRL, Life Technologies, Gaithersburg, MD), penicillin (100 IU/ml), and streptomycin (100 μg/ml) at 37 °C in a humidified chamber containing 5 % CO_2_ incubator. The experiments were performed when the cells reached 80–90 % confluence. In all studies, cells were incubated in the DMEM medium. In certain selective experiments, cells were subsequently incubated in the high glucose (40 mM) medium.

### Experimental protocols

After the cell density reached 70–80 %, the rat vascular smooth muscle cells were divided randomly into five groups: (1) a control group: VSMCs were cultured in 10 % FBS in DMEM; (2) HG + palmitate group: VSMCs were cultured in DMEM with a 40 mM HG and 500 µM palmitate treatment for 24 h; (3) HG + palmitate + NaHS: The procedure was similar to that for group 2, 100 µM NaHS was added in cultured medium for 24 h; (4) HG + Palmitate + NAC: The procedure was similar to that for group 2, 5 µM NAC was added in cultured medium for 24 h; (4) HG + Palmitate + Mdivi-1: The procedure was similar to that for group 2, 50 µM Mdivi-1 was added in cultured medium for 24 h; (5) HG + Palmitate + siRNA Drp1: the procedure was similar to that for group 2, 150 nM siRNA Drp 1 was added in cultured medium for 24 h.

### Cell proliferation assay

HPASMCs were cultured in 96-well tissue culture plates (1 × 10^4^ cells/well) with 10 % FBS for 24 h. Then cells were pretreated with DMEM containing 2 %FBS for 12 h and then treated with different reagents for another 24 h. After treatment with stimuli, cell viability and proliferation were measured by 5-bromo-2′-deoxyuridine (BrdU) incorporation assays. BrdU was observed by a laser confocal scanning microscope. Then cells were analyzed by FACSVerse™ flow cytometer (BD biosciences).

### Scratch wound repair assay

HPASMCs were seeded in 6-well plates and treated with different reagents when the cells reached 80–90 % confluence, and then subjected to the in vitro scratch assay as described previously [17]. Images were captured at 0, 12 and 24 h after treatment using phase-contrast microscopy.

### Measurement of cytosolic and mitochondrial ROS (DCFH, DHE and Mitosox)

To measure cytosolic and mitochondrial ROS production in HPASMCs, cytosolic specific staining with DCFH and DHE and mitochondrial specific staining with Mitosox were used [18]. HPASMCs were incubated in 24-well plates and treated with different reagents for 4 h. Then washed the cells with PBS and incubated with pre-warmed PBS containing at a final working concentration of 10 μM DCFH dye for cytosolic H_2_O_2_ detection at 37 °C for 30 min and 10 μM DHE fluorescent probe for cytosolic superoxide anion (O^2−^) at 37 °C for 1 h. The same process for mitochondrial ROS detection, except incubation in Mitosox at 200 nM. The fluorescence of DCFH was measured using excitation and emission wavelengths of 480 and 535 nm, and the fluorescence of DHE was measured using excitation 510–560 nm and emission of 590 nm, respectively. MitoSOX Red fluorescence was measured at 583 nm following excitation at 488 nm using a Zeiss LSM 510 inverted confocal microscope. Thirty smooth muscle cells in each group were randomly photographed, and their total fluorescence was recorded. Data were analyzed using Flow Version software.

### Vascular smooth muscle cells ultrastructure measurement

Vascular smooth muscle cells immersed immediately in fixative (3.0 % glutaraldehyde buffered in 0.1 M sodium cacodylate, pH 7.2). Following 1–2 days of storage, specimens were raised in PBS, postfixed in cacodylate-buffered 1 % osmium tetroxide, dehydrated in ethanol series, and embedded in polybed 812. Zeiss SUPRA55-VP was used for observation of ultrastructure.

### Mitochondrial fragmentation

Using Mitotracker staining (Invitrogen) to observe the mitochondrial morphology, VSMCs were seeded in 24-well plates and treated with different reagents and 200 nM Mitotracker for 30 min in a 37 °C incubator containing 5 % CO_2_ and then washed with PBS. A confocal laser scanning microscope (LSM 700, Zeiss) was used for visualization and to determine the fluorescence intensity. We subtracted the background from the acquired images; the images were then filtered, and binary operations were applied to identify mitochondria segments using Image J (NIH Bethesda, MD). Continuous mitochondrial structures were counted and the number was normalized to the total mitochondrial area to obtain the mitochondrial fragmentation count (MFC) for each of 25 or more randomly selected cells, as described previously [19]. Cells with greater fragmentation exhibit a higher MFC. The mitochondria lengths were measured using NIS Elements software and scored as follows: fragmented (globular, <2 µm diameter); intermediate (2–4 µm long); and filamentous (>4 µm long). Approximately 200 cells were analyzed, and the experiments were performed in triplicate by two individuals.

### Western blotting

Human renal arteries and rat abdominal aorta were frozen in liquid nitrogen and stored at −80 °C. The tissue was homogenized in RIPA buffer with proteases and phosphates inhibitors. The cells were washed in PBS and lysed for approximately 20 min in lysis buffer (Cell Signaling Technologies) containing 20 mM Tris (pH 7.5), 150 mM NaCl, 1 mM Na_2_-EDTA, 1 mM EGTA, 1 % Triton, 2.5 mM sodium pyrophosphate, 1 mM b-glycerophosphate, 1 mM Na_3_VO_4_, 1 mg/ml leupeptin, and 1 mM phenylmethylsulfonyl fluoride. After the lysate was centrifuged at 3000×*g* for 30 min. The supernatant was collected and stored at −80 °C. The protein concentration was determined using the BCA Protein Assay Kit (Thermo Science). The proteins were separated in a sodium dodecyl sulfate–polyacrylamide gel and transferred to a PVDF membrane (Millipore). After blocking with Tris-buffered saline-0.1 % Tween 20 (TBS-T) containing 5 % nonfat dried milk for 2 h at room temperature, the membrane was washed twice with TBS-T and incubated with a primary antibody [CSE (42 kDa), Cyclin D1 (36 kDa), P27 (27 kDa), PCNA(36 kDa), P21 (21 kDa), Drp (17 kDa), Mfn-2 (86 kDa), α-smooth muscle actin (42 kDa), MMP2 (72 kDa), MMP9 (67–92 kDa), Collagen-I (129 kDa) and Collagen-Ш (140 kDa) proteins] overnight at 4 °C. The membrane was washed three times with TBS-T for 10 min and then incubated for 2 h at room temperature with horseradish peroxidase-conjugated secondary antibodies. The bands were detected using an enhanced chemiluminescence (ECL) reagent (Santa Cruz Biotechnology). Densitometry was carried out with image processing and analysis program Image J 1.48 and the data were expressed as relative units. The band normalized against actin.

### Transfection with a small interfering RNA (siRNA)

Predesigned Drp1-targeted siRNA (Drp-1-siRNA), a pool of three target-specific 19 to 25-siRNA designed to knock down Drp-1 gene expression was purchased from Santa Cruz. Transfection of SMCs by siRNA was achieved using lipofectamine 2000. In brief, Drp-1 siRNA and the transfection reagent were incubated for 20 min to form complexes, which then were added to plates containing cells and medium. The cells were incubated at 37 °C in a CO_2_ incubator for further analysis.

### Statistical analysis

The quantified data are the average of at least triplicate samples. The error bars represent standard errors of the mean. More than two groups were compared using a one-way ANOVA and Bonferroni’s correction. Differences between individual groups were analyzed using Student’s *t* test. A P value less than 0.05 was considered as significant.

## Results

### H_2_S levels and associated proliferation protein expression levels in human renal arteries from type 2 diabetes patients and db/db mice

**Fig. 1:**
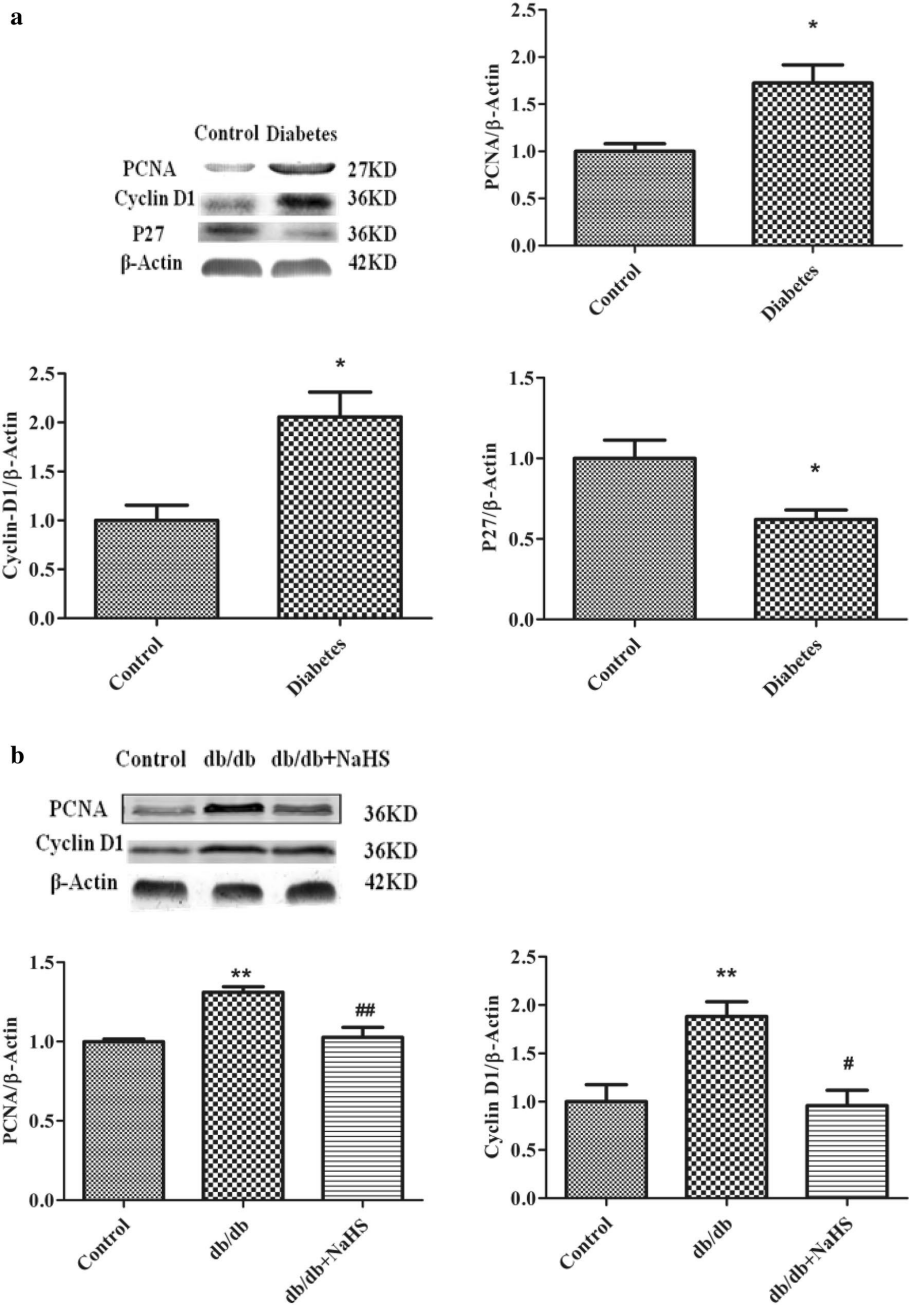
Cell proliferative protein expression levels in human renal arteries and aorta from db/db mice. PCNA, Cyclin D1 and P27 expression levels in renal arteries from type 2 diabetes patients (**a**) and in aorta from the control mice, db/db mice and db/db mice treated with NaHS (**b**) (*p < 0.05 vs control, **p < 0.01 vs control, ^#^ p < 0.05 vs db/db mice, and ^##^ p < 0.01 vs db/db mice)

Some studies have demonstrated that chronic hyperglycemia has been shown to stimulate HPASMC proliferation and migration [19]. At first, we demonstrated that blood glucose levels of db/db mice were routinely above 20 mM (Additional file 1: Figure S1). In our study, we found that PCNA and Cyclin D1 protein expression increased, whereas P27 expression decreased in renal arteries from type 2 diabetes patients (Fig. 1a). As the similar results, PCNA and Cyclin D1 expression levels were markedly greater in db/db mice than control group (Fig. 1b).

**Fig. 2:**
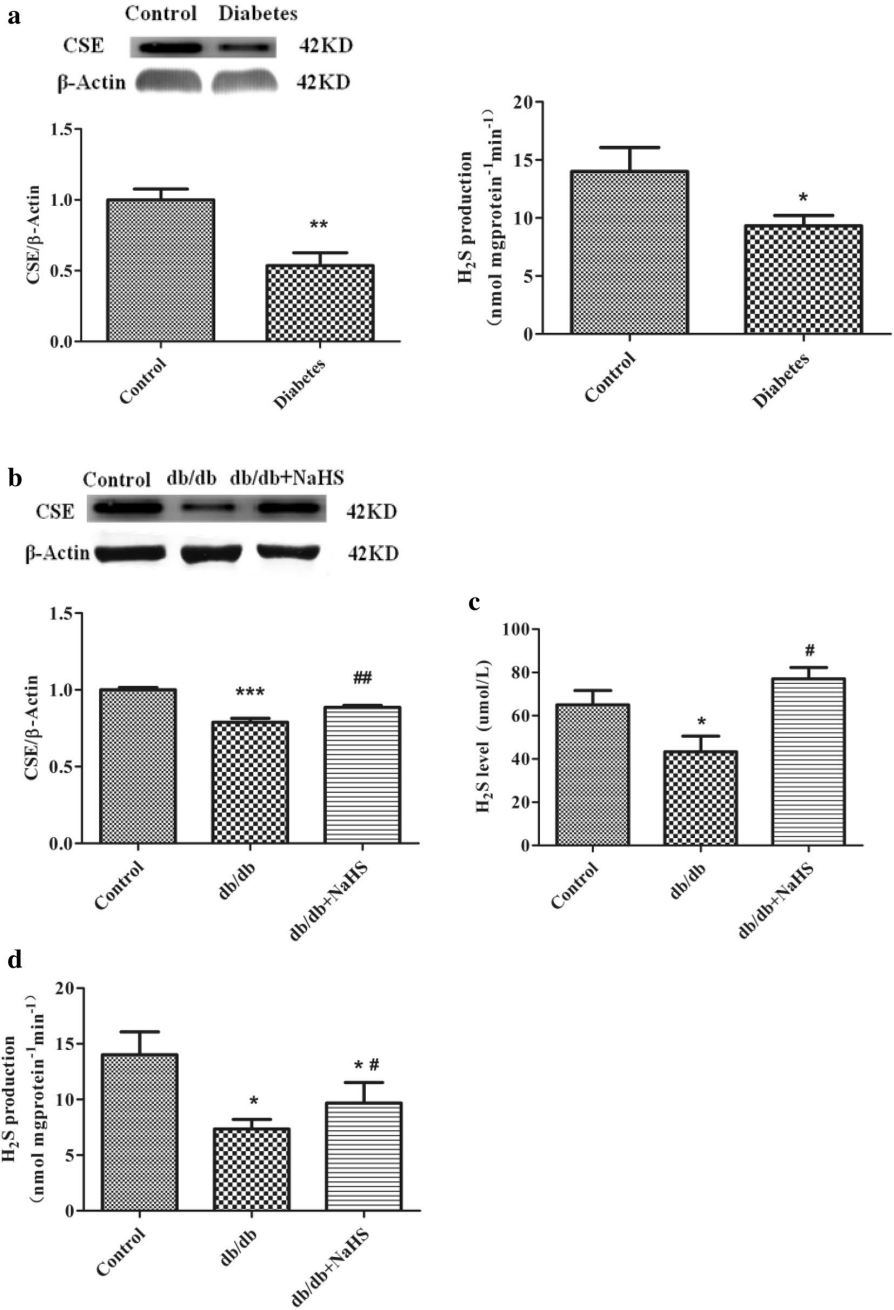
H_2_S levels and CSE expression levels in human renal arteries and aorta from db/db mice. CSE expression and H_2_S production rate (**a**) from type 2 diabetes patients. CSE expression (**b**) and H_2_S level (**c**) and H_2_S production rate (**d**) in aorta from the control mice, db/db mice and db/db mice treated with NaHS. (n = 4–6 in each group) (*p < 0.05 vs control, **p < 0.01 vs control, ^#^ p < 0.05 vs db/db mice, and ^##^ p < 0.01 vs db/db mice)

Yang et al. [15] have revealed that CSE knock-out mice stimulated vascular smooth muscle cells proliferation. Our previous study showed that CSE expression in type I diabetes is lower [20]. To further investigate whether chronic hyperglycemia affects CSE expression in aorta arteries from db/db mice, CSE expression and H_2_S levels were detected. CSE expression in renal arteries from type 2 diabetes patients (Fig. 2a) and in aortic arteries from db/db mice (Fig. 2b) were lower compared with non-diabetes patients and control and db/db-NaHS mice. The db/db mouse plasma H_2_S concentrations were also lower than in control mice and in mice treated with NaHS (Fig. 2c). Simultaneously, the level of H_2_S synthase CSE activity was also detected in the aortic arteries of db/db mice. Our data show that H_2_S production rate was also significantly lower in type 2 diabetes patients and db/db mice compared with that of non-diabetes patients and the control and db/db-NaHS mice (Fig. 2d).

### Exogenous H_2_S inhibiting HG and palmitate-stimulated VSMC proliferation and migration

**Fig. 3:**
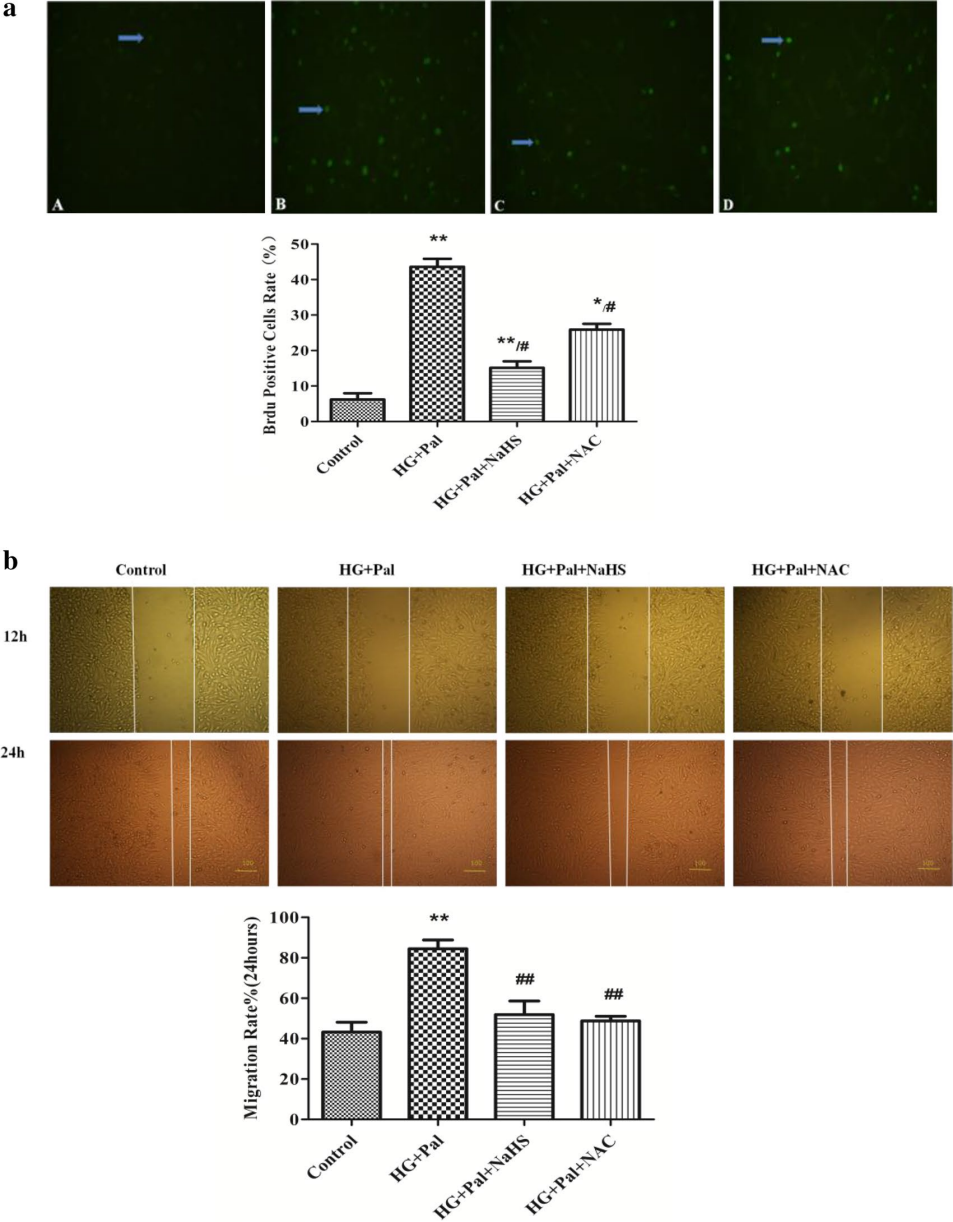
NaHS inhibits high glucose (HG)-induced VSMC proliferation and migration. **a** After HG stimulation for 48 h, cell proliferation was determined using the BrdU assay (n = 4). **b** After stimulation with HG and palmitate for 12 and 24 h, migration assays were performed. The results are representative of three independent experiments. *p < 0.05 vs control, **p < 0.01 vs control, ^#^ p < 0.05 vs HG and Pal, ^##^ p < 0.01 vs HG and Pal

To investigate whether exogenous H_2_S affects HG and palmitate-mediated HPASMC proliferation and migration enhancement, three separate experiments were performed. As shown in Fig. 3a, VSMCs stimulated with 40 mM HG and 500 μM palmitate for 24 h exhibited a significant increase in proliferation rate compared with control group using the BrdU assay. The increase in proliferation rate was attenuated by NaHS treatment.

Using the scratch assay, HG and palmitate significantly enhanced the HPASMC migration rates of two and threefolds for 12 and 24 h, respectively, compared with control groups. 100 μM NaHS pretreatment prevented theses increases in cells exposed to HG and palmitate (Fig. 3b).

### Exogenous H_2_S inhibiting mitochondrial oxidative stress due to high glucose and palmitate in HPASMC s

**Fig. 4:**
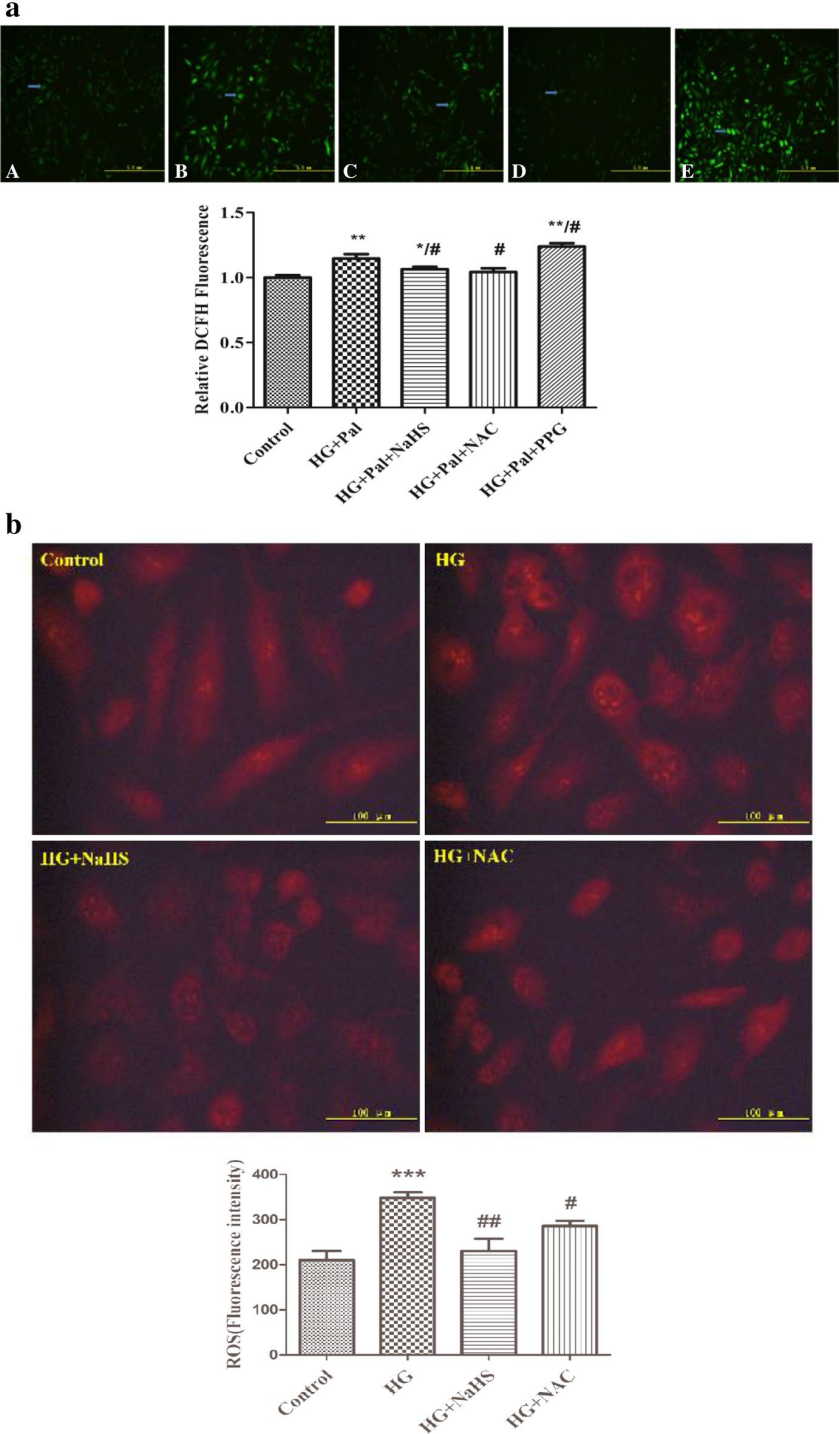
NaHS inhibits high glucose (HG)-induced VSMC ROS production. Cytoplasm H_2_O_2_ (**a**) and superoxide anion (**b**). *p < 0.05 vs control, **p < 0.01 vs control, ^#^ p < 0.05 vs HG and Pal, ^##^ p < 0.01 vs HG and Pal

To test whether exogenous H_2_S decreases ROS production in cultured smooth muscle cells, we used a DCFH fluorescent probe. As shown in Fig. 4a and b, the high glucose and palmitate treatment significantly increased ROS levels compared with HPASMC s in the control culture medium. Further, a 100 μM NaHS treatment decreased the ROS levels induced by HG and palmitate in HPASMCs. DL-proparglycine (PPG), an irreversible competitive CSE inhibitor, was confirmed the role of H_2_S in reducing ROS level. Our data showed that ROS level in PPG group is significantly higher than that in NaHS group (Fig. 4a).

**Fig. 5:**
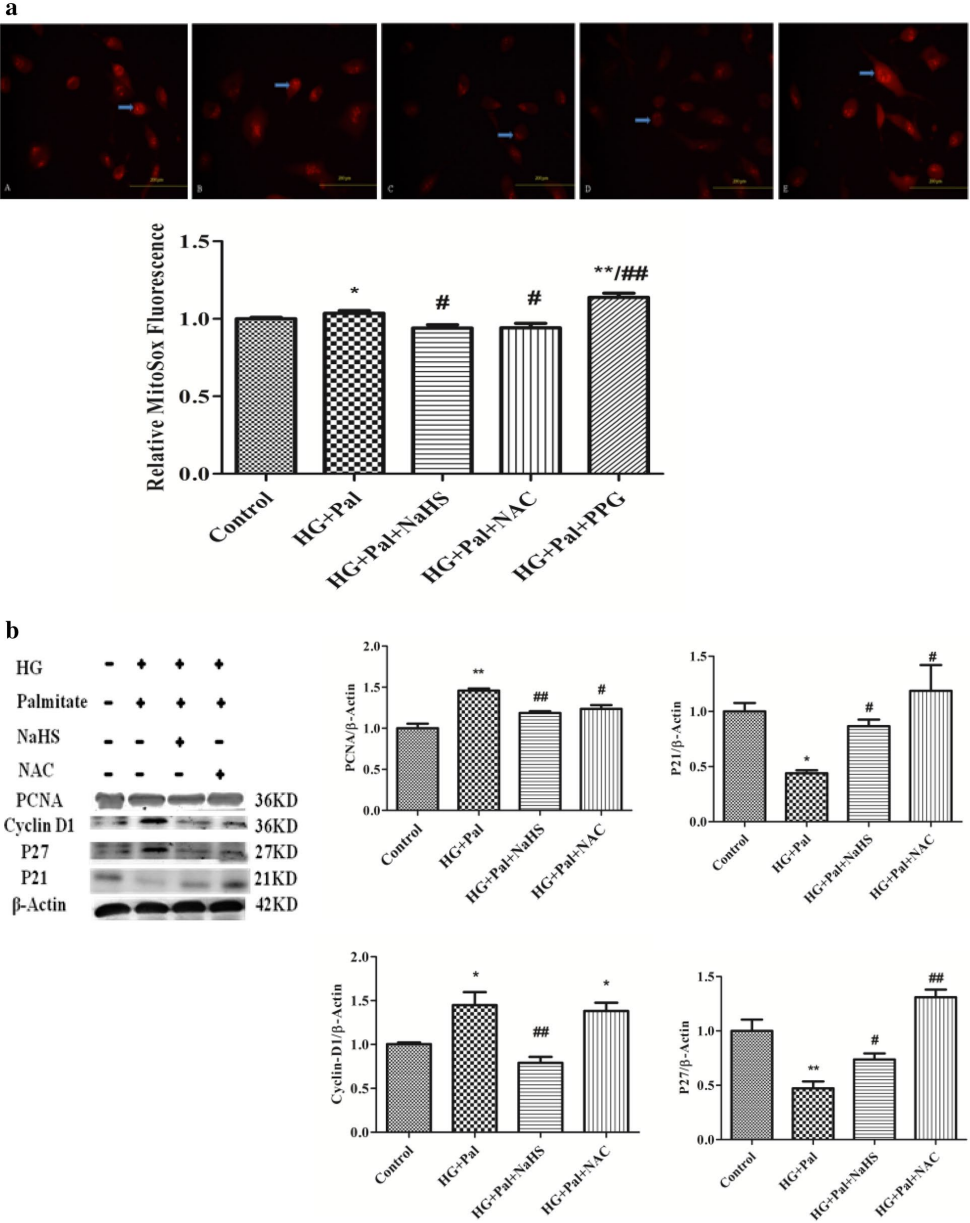
NaHS inhibits high glucose (HG)-induced VSMC cell proliferative protein expression. Mitochondrial (**a**) ROS production were assessed using 5 µM MitoSox. Representative fluorescence images show increased ROS production in the VSMC mitochondria. **b** Upon stimulation with HG for 24 h, the P27, PCNA and Cyclin D1 protein expression levels were determined using western blot analyses; the results are representative of three independent experiments. *p < 0.05 vs control, **p < 0.01 vs control, ^#^ p < 0.05 vs HG and Pal, ^##^ p < 0.01 vs HG and Pal

MitoSox is a ROS sensitive probe that can detect ROS production in living cell mitochondria. Thus, we used MitoSox to label ROS and monitor ROS changes in SMCs with various treatments. ROS levels are positively related to MitoSox fluorescence emission intensities. As shown in Fig. 5a, the cells treated with HG and Pal or treated with HG and Pal and PPG showed a significant increase in MitoSox fluorescence immediately after treatment, which is in contrast to the slight increase observed in the control cells and cells treated with exogenous H_2_S and NAC.

### The effect of exogenous H_2_S on cell cycle regulatory proteins’ MMPs and collagen expression in HPASMCs

We determined the level of cell cycle regulatory protein expression using western blot analyses. The Cyclin D1 and PCNA expression levels were elevated within 24 h in the presence of HG and palmitate. However, the P27 and P21 levels decreased. For VSMCs treated with 100 μM NaHS, the Cyclin D1 and PCNA expression levels decreased within 24 h, and the P27 and P21 levels increased (Fig. 5b).

**Fig. 6:**
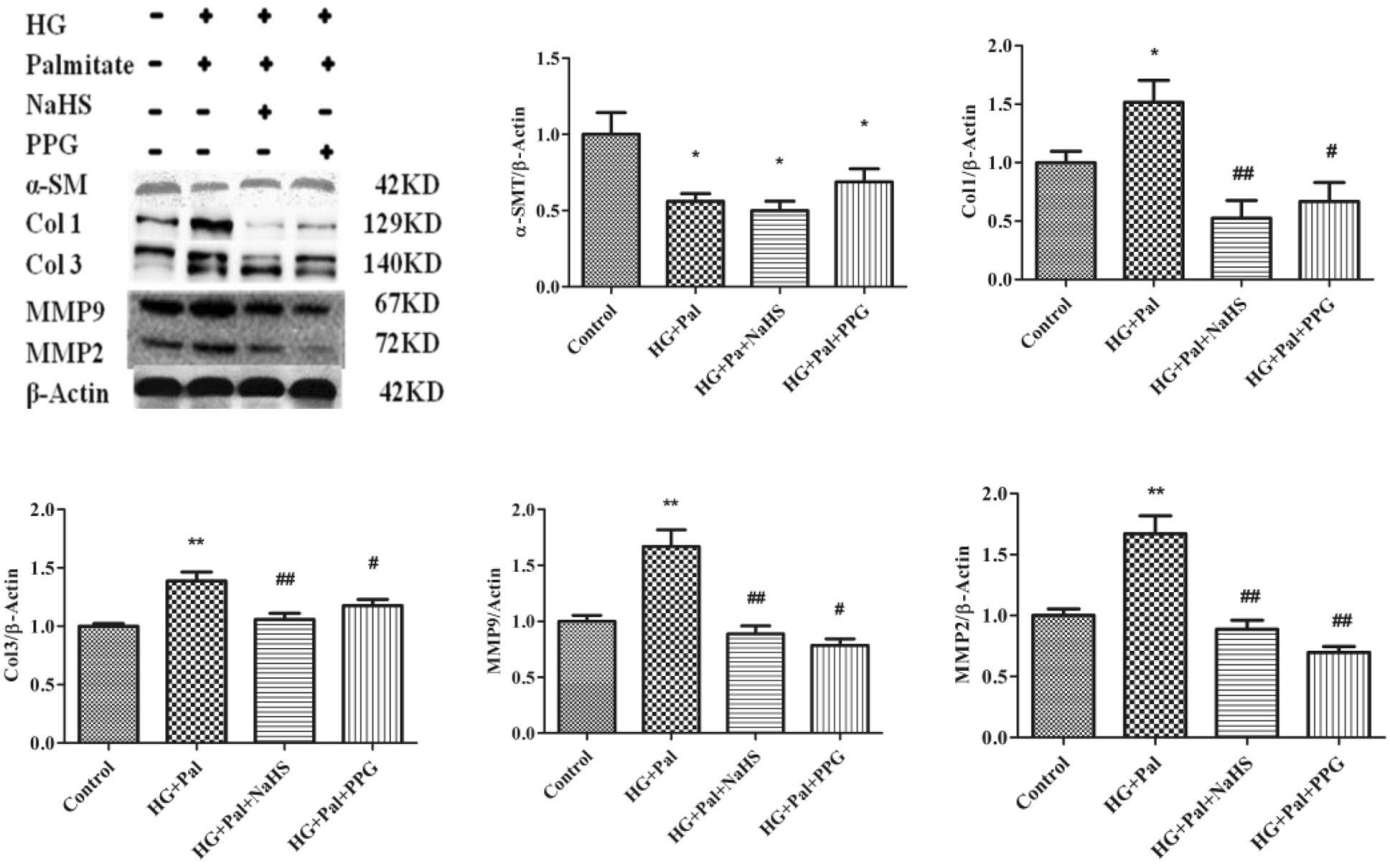
NaHS inhibits high glucose (HG)-induced cell proliferative protein expression. Cells stimulated with HG for 24 h. The protein expression levels of α-SM, collagen I, collagen Ш, MMP9 and MMP2 were determined using western blot analyses (n = 3). *p < 0.05 vs control, **p < 0.01 vs control, ^#^ p < 0.05 vs HG and Pal, ^##^ p < 0.01 vs HG and Pal

Oxidative stress regulates MMPs, which play a role in collagen remodeling of the matrix in hyperglycemia [21, 22]. Therefore, we measured the MMP-2 and MMP-9 expression levels in cultured VSMCs using western blotting. Our data showed that the MMP-2 and MMP-9 expression levels significantly increased in the HG and palmitate and PPG groups. H_2_S inhibited expression of both proteins in HPASMCs. Collagen is a major component of the extracellular matrix, and excessive collagen accumulation is related to tissue remodeling. The results showed that the HPASMC collagen I and collagen Ш expression levels significantly increased in the HG and palmitate and PPG groups. H_2_S could ameliorate expression of both proteins in HPASMCs (Fig. 6).

### Mitochondrial fission/fusion-associated protein expression levels in kidney arteries from type 2 diabetes patients and aortic arteries from db/db mice

Mitofusion initiates the assembly of individual mitochondria that combine their membranes. This process is regulated by mitofusion (Mfn) 1 and 2 which are evolutionarily conserved GTPase proteins that attach to Outer Mitochondrial Membranes (OMM) [23]. During mitochondrial fusion, Mfn 1 and 2 promote rearrangement of mitochondrial membranes [24, 25]. More and more evidence has demonstrated that Mfn 2 plays the key role in controlling mitochondrial fusion [26].

**Fig. 7:**
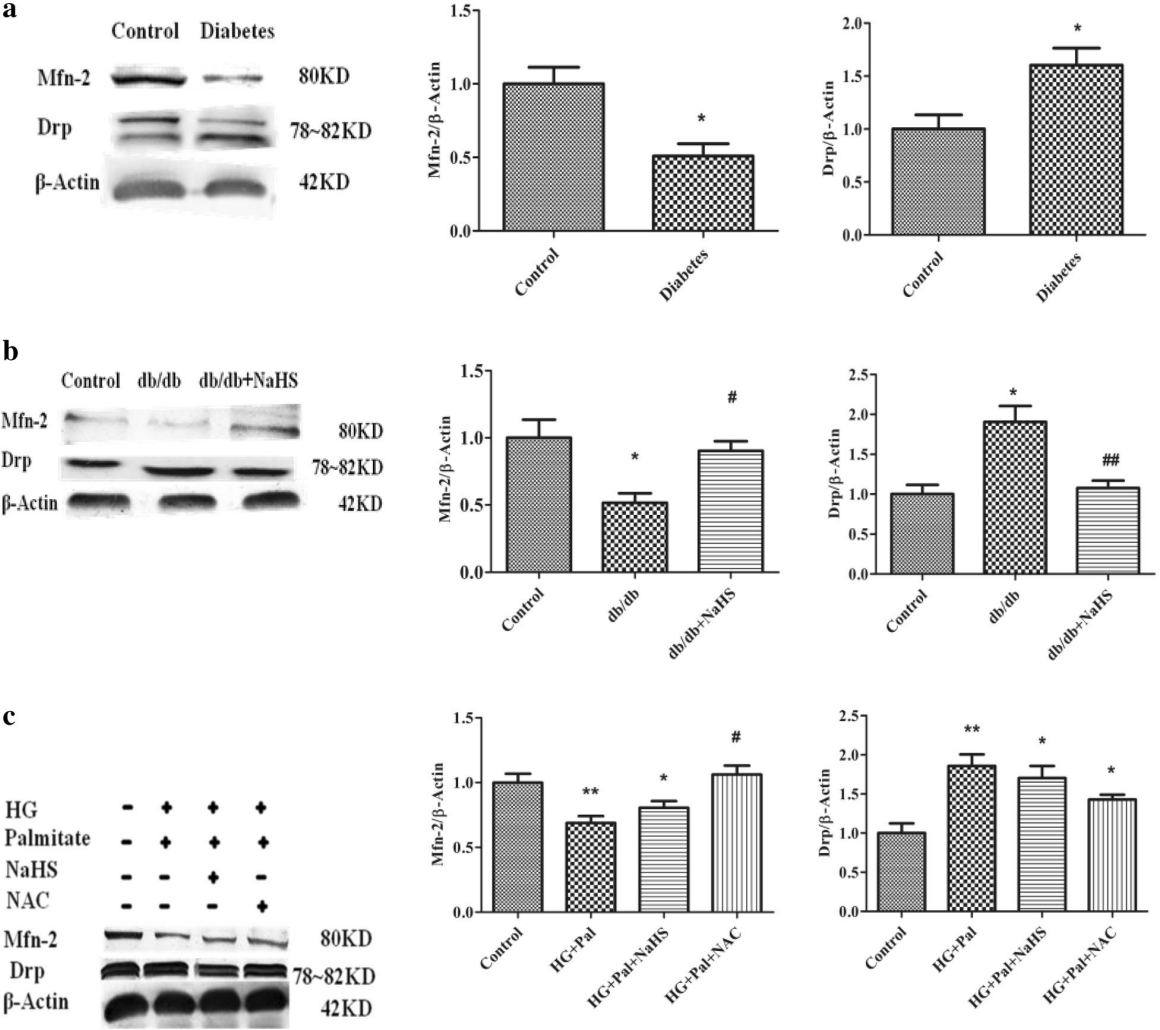
Mfn-2 and Drp protein expression in renal arteries from type 2 diabetes patients (**a**) and in db/db mice (**b**) and in cultured HPASMC treated with HG and palmitate (**c**). (n = 3–5 in each group) *p < 0.05 vs control, **p < 0.01 vs control, ^#^ p < 0.05 vs HG and Pal, and ^##^ p < 0.01 vs HG and Pal

To identify the arterial smooth muscle cell proliferation machinery in mitochondria dynamics, we determined the expression levels of the mitochondrial dynamic proteins Drp1 and Mfn-2. The Drp1 expression increased in renal arteries of type 2 diabetes patients and in aorta of db/db mice compared with that in the control patients and mice treated with exogenous H_2_S, whereas the Mfn-2 expression decreased in kidney arteries from patients with type 2 diabetes and aortic arteries from db/db mice compared with that in the control patients and mice treated with exogenous H_2_S (Fig. 7a, b).

### NaHS inhibited mitochondria fission to prevent HPASMC hyperproliferation

Studies have demonstrated that mitochondrial fission increases oxidation metabolism in pulmonary arterial hypertension (PAH) [27]. Drp1 mediated mitochondrial fission in PAH. We examined the Mfn-2 and Drp1 expression levels using western blot analyses. We found that the expression of Mfn-2 decreased, and the expression of Drp1 increased in VSMCs treated with HG and palmitate compared with that in VSMCs treated with exogenous H_2_S and NAC (Fig. 7c). To further investigate that exogenous H_2_S could modulate mitochondrial fusion and fission, we detected the expression of Mfn 2 and Drp-1 in mitochondria. Our data revealed that high glucose and palmitate reduced the expression of Mfn 2 and promoted the expression of Drp-1, whereas, exogenous H_2_S enhanced the expression of Mfn 2 and down-regulated the expression of Drp-1 in mitochondria (Additional file 1: Figure S2 A, B).

### Mitochondrial fragmentation due to oxidative stress caused by high glucose and palmitate

**Fig. 8:**
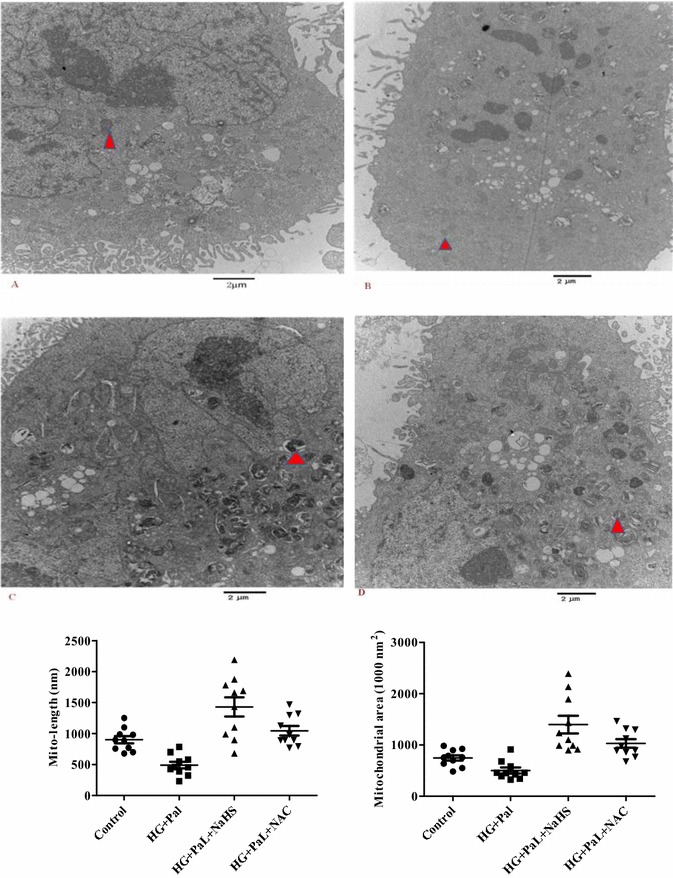
Representative mitochondrial morphology in VSMCs observed using electron microscopy (EM). *Scale bar* at high magnification = 2 µm. *p < 0.05 vs control, **p < 0.01 vs control, ^#^ p < 0.05 vs HG and Pal, and ^##^ p < 0.01 vs HG and Pal

To further investigate mitochondrial fragmentation, mitochondrial morphology was observed by transmission electron microscopy (TEM). Most control cells showed normal and tubular mitochondria. In contract, HG-pal-treated cells exhibited mitochondria with a fragmented, punctiform morphology, whereas H_2_S-treated SMCs exhibited normal and tubular mitochondria. TEM confirmed that HG and palmitate induced mitochondrial fragmentation in VSMCs compared with the control and NaHS groups (Fig. 8a).

### Exogenous H_2_S inhibited VSMCs proliferation through down regulating Drp1

**Fig. 9:**
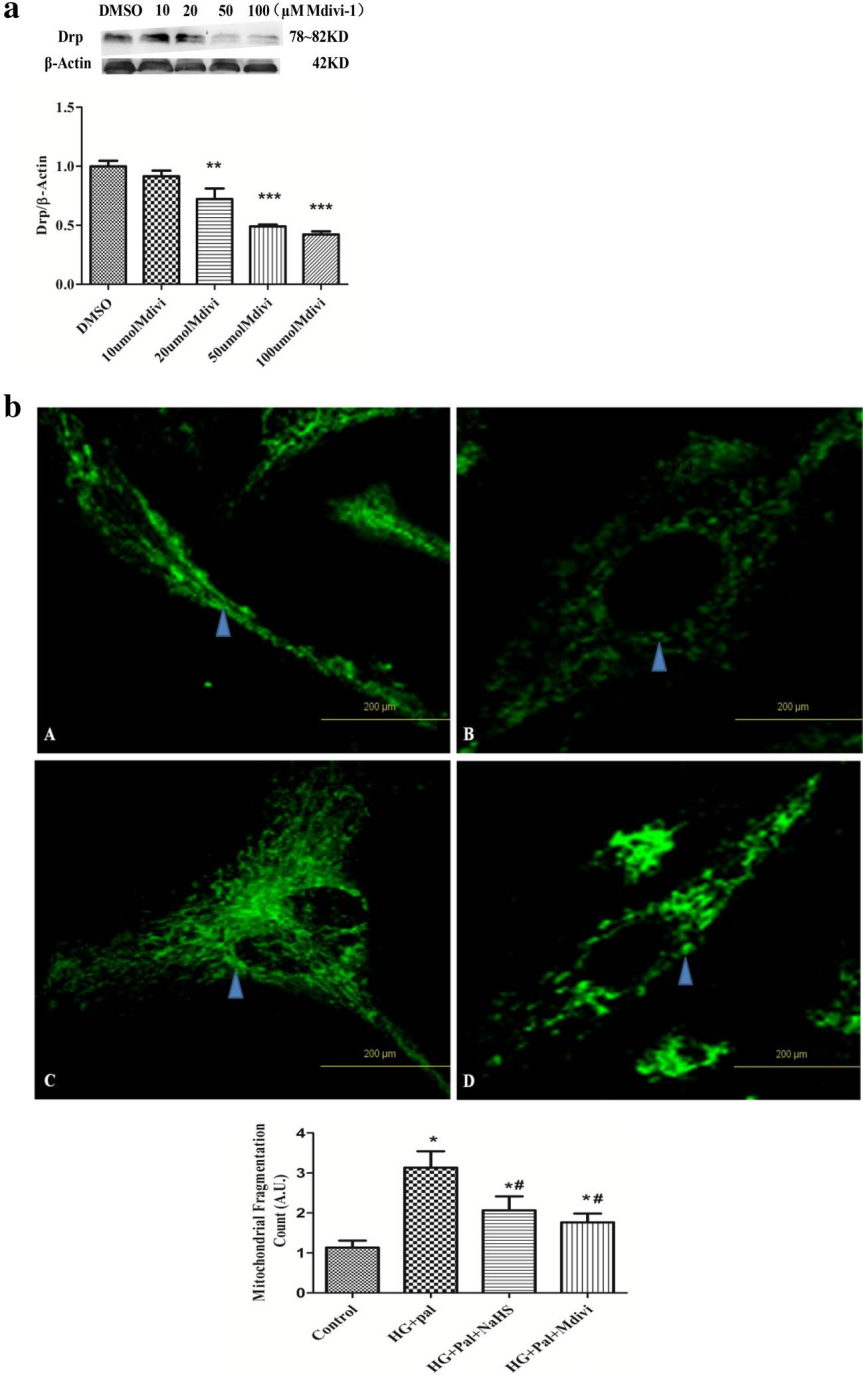
Exogenous H_2_S and Mdivi-1 inhibit mitochondrial fragmentation to reduce proliferation in VSMCs. **a** Different concentrations of Mdivi-1 affected Drp expression. (n = 4) (**p < 0.01 vs DMSO, ***p < 0.001 vs DMSO). **b** Increased mitochondrial fragmentation count in VSMCs with the HG and palmitate treatment compared with VSMCs treated with NaHS and Mdivi-1. The results are representative of three independent experiments (*p < 0.05 vs control, ^#^ p < 0.05 vs HG and Pal)

The small molecule inhibitor of Drp1, mitochondrial division inhibitor (Mdivi-1), was used to inhibit Drp expression in a dose-dependent manner (Fig. 9a). The expression of Drp was decreased with the pre-administration of Mdivi-1 in a dose-dependent way. SMCs were transiently transfected with Mitotracker to determine the mitochondrial fragmentation. We also found that the increase of mitochondrial fragmentation depended on Drp1 upon treatment with HG and palmitate (Fig. 9b).

**Fig. 10:**
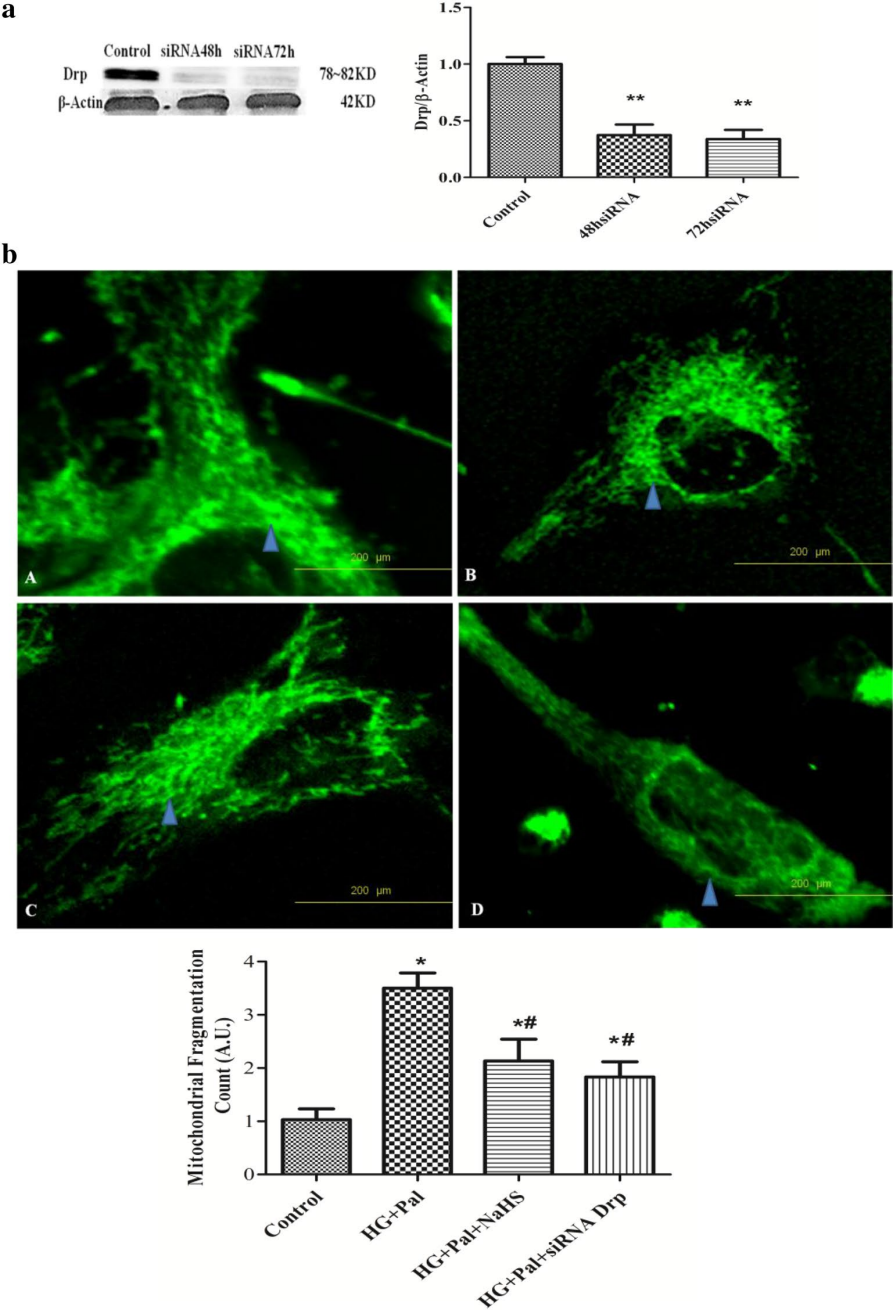
Silencing Drp1 expression prevents HG-induced ROS production and migration as well as cell proliferative protein expression. **a** siRNA transfection effectively reduced Drp1 expression. (n = 3) ROS production in the cytoplasm (**b**) *p < 0.05 vs control, ^#^p < 0.05 vs HG and Pal, and ^##^p < 0.01 vs HG and Pal

**Fig. 11:**
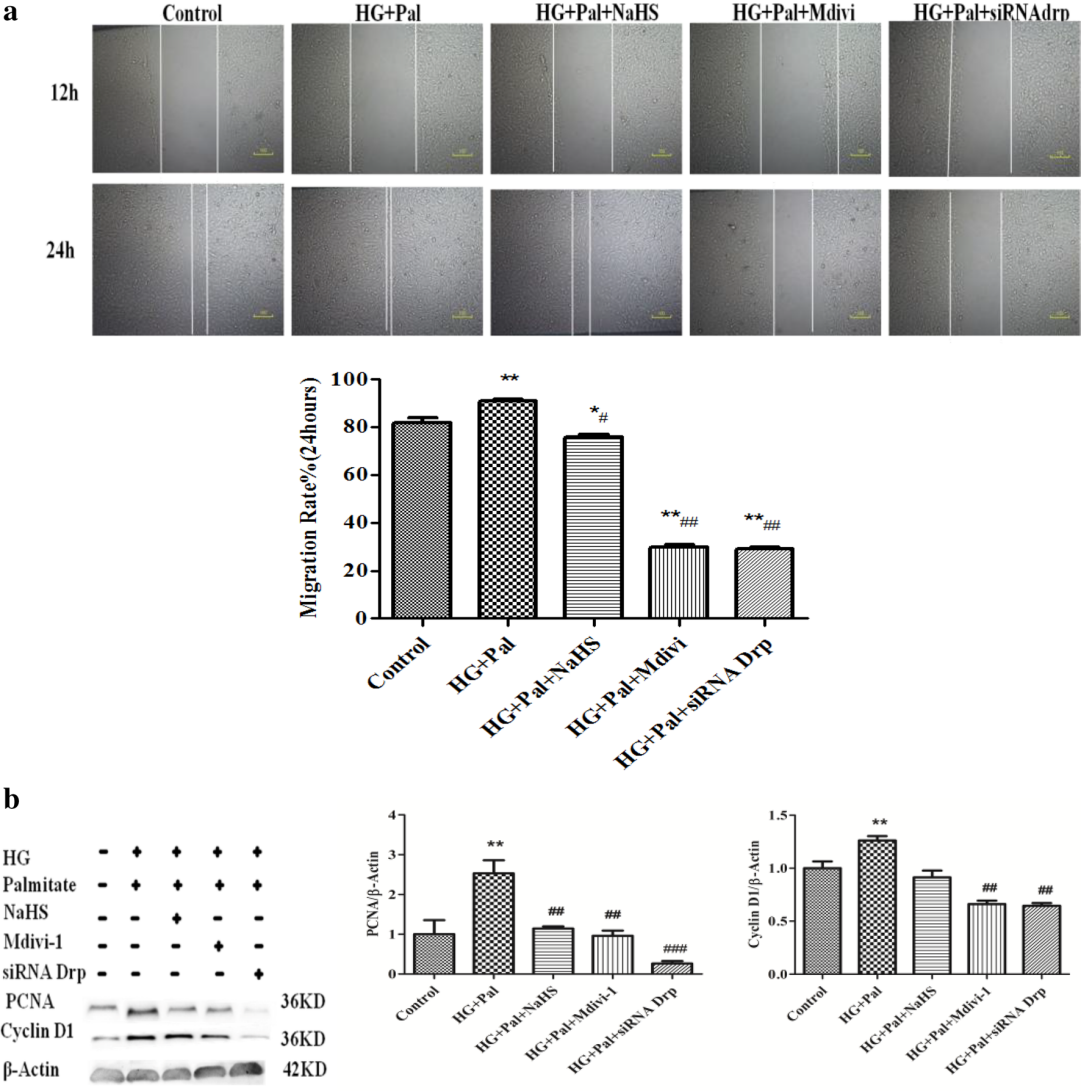
Silencing Drp1 expression prevents HG-induced ROS production and migration as well as cell proliferative protein expression. **a** Silencing Drp1 expression inhibits the HG-induced increase in mitochondrial fragmentation count. (The data are the mean ± SD for 4 experiments). **b** siRNA Drp1 reduced PCNA and Cyclin D1 protein expression and increased P27 and P21 protein expression (n = 4). *p < 0.05 vs control, ^#^p < 0.05 vs HG and Pal, and ^##^p < 0.01 vs HG and Pal

To further explore the role of Drp1 in mitochondrial fission, we knocked down Drp1 expression with siRNA. Drp1 siRNA decreased Drp1 protein expression after transfection for 72 h (Fig. 10a) and inhibited mitochondrial fragmentation in VSMCs (Fig. 10b). As shown in Fig. 11a, Mdivi-1 and Drp1 siRNA also exhibited anti-migration effects upon treatment with HG and palmitate. Drp1 siRNA inhibited the HPASMC migration rate as well as PCNA and Cyclin D1 protein expression.

To further determine whether inhibition of mitochondria fission by transfecting siRNA Drp-1 and Mdivi-1 would effect on HPASMCs proliferation phenotype, we detected HPASMCs proliferation rate using BrdU assay and the expression of Collagen I,III and MMP2,9. Our results revealed that Mdivi-1 and siRNA Drp-1 and NaHS reduced the proliferation rate in HPASMCs compared with HG and Pal group (Additional file 1: Figure S3A). Simultaneously, siRNA Drp-1 and NaHS decreased the expression of Collagen I,III and MMP2,9 compared with HG and Pal group (Additional file 1: Figure S3B).

## Discussion

VSMC proliferation in the arterial wall plays an important role in forming in-stent restenosis in diabetic humans. VSMC proliferation contributes, in part, to increased oxidative stress [28]. Studies have demonstrated that ROS generation may promote VSMC proliferation-induced vascular injury in diabetes patients. Recent studies report that mitochondrial fission is a mitotic checkpoint that allows cells to proliferate rapidly. Inhibiting mitotic fission using Mdivi-1 fuses mitochondria and traps cells in the G2/M phase of the cell cycle, which slows proliferation and promotes apoptosis [29]. In this study, we demonstrate for the first time that H_2_S attenuates HG and palmitate-induced HPASMC proliferation by inhibiting mitochondrial fragmentation. Likely, H_2_S affects mitochondrial dynamics through differential modulation of mitochondrial fission and fusion proteins, which impairs the mitochondrial fusion–fission balance because manipulating Drp1 and Mfn2 expression altered the effects of H_2_S.

Several studies show that vascular reactive oxygen production is related to NADPH oxidases, xanthine oxidase, lipoxygenase, and mitochondrial election transport. Considering the ROS-induced cell-proliferation mechanism, a growing body of evidence suggests that ROS play a vital role in activating MAPK and PI3K/Akt cascades mediated by HG [30]. Over the past decade, several investigators have shown that H_2_O_2_ and ROS regulate cell proliferation. Transient H_2_O_2_ production is considered an intracellular signal for cell growth and transformation triggered by surface receptor activation and determined by mitochondrial metabolic status [31]. Our results showed that HG increased ROS in the mitochondria and cytoplasm, whereas exogenous H_2_S reduced ROS production.

The major observation from this study is that hyperglycemia and high glucose-induced oxidative stress caused mitochondrial fragmentation in VSMCs, which was demonstrated by confocal microscopic imaging and TEM. Because mitochondrial morphology is tightly controlled by the balance between mitochondrial fission and fusion [32]. We postulate that hyperglycemia and high glucose-induced mitochondrial fragmentation is caused by enhancing fission and reducing fusion. To support this notion, we used the mitochondria-targeted fluorescent probe mitotracker to demonstrate that mitochondria in high glucose-treated cells were nearly unable to fuse compared with control mitochondria. However, exogenous H_2_S also inhibited mitochondrial fission and promoted mitochondrial fusion. At the molecular level, we observed that high glucose-triggered ROS activated Drp-1, which was indicated by a significantly increased association with mitochondria. Exogenous H_2_S may inhibit Drp-1 mitochondrial translocation primarily through changes in mitochondrial and cytosolic ROS. Drp1 activation may contribute to the increased fission rate in our models.

Changes in mitochondrial architecture during the cell cycle due to fission and fusion events have been observed [32, 33]. Ryan et al. demonstrated that inhibiting dynamin-related protein function (using Midivi-1) inhibited cell cycle progression and reduced cell proliferation rates in cells cultured form human pulmonary arteries [34]. Studies show in mitochondrial morphology that have not been previously reported during cell proliferation [35, 36]. We used siRNA for the mitochondrial fission inhibitor Mdivi-1 and dynamin-related protein (Drp1) to decrease mitochondria dynamics. The results from treatment with Drp1 siRNA and Mdivi-1 also suggest that mitochondrial fission is required for cell division.

Taken together, the findings presented here suggest that high glucose-induced VSMC proliferation is involved in abnormal mitochondrial dynamics. The data indicate that exogenous H_2_S inhibits mitochondrial fragmentation and reduces VSMC proliferation with high glucose and palmitate treatments. Given the study on mitochondrial fragmentation in VSMCs, exogenous H_2_S likely modulates Drp 1 expression.

## Additional file

10.1186/s13578-016-0102-x The effect of exogenous H_2_S on glucose homeostasis in db/db mice. **Figure S2.** The expression and localization of Mfn 2 and Drp-1 in mitochondria of HPASMCs treated with high glucose and palmitate. **Figure S3.** NaHS and siRNA Drp-1 regulating HPASMCs proliferation rate and phenotype.

## Abbreviations

CSE: cystathionine gamma-lyase
Drp 1: dynamin-related protein 1
H_2_S: hydrogen sulfide
HG: high glucose
HPASMC: vascular smooth muscle cells of human pulmonary aorta
Mdivi-1: mitochondrial division inhibitor 1
Mfn-2: mitofusion 2
MMP-2,-9: matrix metalloproteinase-2,-9
NAC: *N*-acetyl-cysteine
Pal: palmitate
PCNA: proliferative cell nuclear antigen
PPG: DL-proparglycine
ROS: reactive oxygen species
VSMC: vascular smooth muscle cell
