# Supplementary material for: Exogenous H2S modulates mitochondrial fusion–fission to inhibit vascular smooth muscle cell proliferation in a hyperglycemic state
Source: Cell Biosci. 2016 May 31;6:36. doi: 10.1186/s13578-016-0102-x (PMC4888644; doi:10.1186/s13578-016-0102-x)

**Supplement**

**Exogenous H_2_S modulates mitochondrial fusion-fission to inhibit vascular smooth muscle cell proliferation in a hyperglycemic state**

**Methods:**

**Weight Measurement**

Take the mice in the electronic weight meter on a fixed time every week to determine the weight and record values.

**Blood Glucose**

A fixed time every week with a sterility scissors tail edge in mice, using electronic test their blood glucose levels and record values.

**Glucose Tolerance Test**

Mice celiac injection of glucose ( 2g/kg), and collection the blood in 20min, 40min, 60min, 80min, 100min and120min in tail to use electronic glucose meter measuring blood glucose.

**Separation of mitochondrial protein from HPASMCs**

Isolation of mitochondria protein from HPASMCs was performed according to the manufacturer’s protocol (Beyotime, Nantong, China). HPASMCs (n = 4, per group) were washed twice with ice-cold PBS, resuspended in lysis buffer (mM: 20 Hepes/KOH, pH 7.5, 10 KCl, 1.5 MgCl_2_, 1.0 sodium EDTA, 1.0 sodium EGTA, 1.0 dithiothreitol, 0.1 PMSF, and 250 sucrose), and then homogenized by an homogenizer in ice/water. After removing the nuclei and cell debris by centrifugation at 1000g for 10 min at 4 ˚C, the supernatants were further centrifuged at 10000g for 10 min at 4 ˚C. The resulting mitochondrial pellets were resuspended in lysis buffer. The supernatants from the 10000g centrifugation were centrifuged once more at 100000g for 1 h at 4 ˚C and then collected. Mitochondrial fractions were stored at -80 ˚C. Proteins from mitochondria were used to measure the protein level of Mfn 2 and Drp-1.

**Immunofluorescence assay**

For immunofluorescence microscopy, cells were grown on glass coverslips and then fixed for 15 min at room temperature with pre-warmed 4% PFA. Cells were permeabilized for 5 min with 1% Triton X-100 in PBS and incubated with primary antibodies. After incubation with Alexa Fluor– conjugated secondary antibodies (Invitrogen), images were acquired with a 63× oil immersion objective on a confocal microscope (Radiance 2000; Bio-Rad Laboratories).

**Statistical analysis**

The quantified data are the average of at least triplicate samples. The error bars represent standard errors of the mean. More than two groups were compared using a one-way ANOVA and Bonferroni's correction. Differences between individual groups were analyzed using Student’s *t* test. A P value less than 0.05 was considered as significant.

**Results:**

**1. Assessing body weight and hyperglycemia levels in db/db mice and in mice with an exogenous H_2_S treatment.**

At 18 weeks of age, the db/db mouse body weights were significantly greater than normal mice (58.6±2.2, 56.7±3.6, and 21.2±2.7 g) (Fig.1 A), and blood glucose levels of the db/db mouse were significantly higher than in the control mice (Fig.1 B.C, p<0.05).

**2. The expression of Mfn 2 and Drp-1 in mitochondria of HPASMCs**

We examined the expression of Mfn 2 and Drp-1 in mitochondria of HPASMCs with the treatment of high glucose and palmitate. Our data revealed that high glucose and palmitate reduced the expression of Mfn 2 and promoted the expression of Drp-1, whereas, exogenous H_2_S enhanced the expression of Mfn 2 and down-regulated the expression of Drp-1 in mitochondria (Fig.2.A).

To further elucidate the mechanism of high glucose and palmitate regulating mitochondrial morphology, changes in Mfn 2 and Drp-1 levels and localization were assessed by immunofluorescence microscopy . Of note, mitochondrial localization of the fission protein Drp-1 significantly increased in HPASMCs after high glucose and palmitate treatment compared with that in HPASMCs treated by NaHS and NAC (Fig. 2.B) ; whereas, the localization of Mfn 2 significantly decreased in HPASMCs after high glucose and palmitate treatment compared with that in HPASMCs treated by NaHS and NAC (Fig. 2.C).

**3. Inhibition of Drp-1 effecting on HPASMCs proliferation phenotype**

To further determine whether inhibition of mitochondria fission by transfecting siRNA Drp-1 and Mdivi-1 would effect on HPASMCs proliferation phenotype, we detected HPASMCs proliferation rate using BrdU assay and the expression of Collagen I, III and MMP2, 9. Our results revealed that Mdivi-1 and siRNA Drp-1 and NaHS reduced the proliferation rate in HPASMCs compared with HG and Pal group (Fig.3A). Simultaneously, siRNA Drp-1 and NaHS decreased the expression of Collagen I, III and MMP2, 9 compared with HG and Pal group(Fig.3B).

**Figure 1 The effect of exogenous H_2_S on glucose homeostasis in db/db mice. (A)** Body weights for the control mice, db/db mice and db/db mice treated with NaHS. **(B)** Blood glucose levels of control (untreated, non-diabetic mice), db/db mice and db/db mice treated with NaHS for 8 weeks. **(C)** The glucose tolerance test was performed in the control mice, db/db mice and db/db mice treated with NaHS after 8 weeks of treatment. Blood glucose concentrations for the control treated and untreated mice at 0, 30, 60, 90 and 120 min after an i.p. glucose injection. (***P<0.001 vs control, #P<0.05 vs db/db mice).

**Figure 2. The expression and localization of Mfn 2 and Drp-1 in mitochondria of HPASMCs treated with high glucose and palmitate. (A)** The expression of Mfn 2 and Drp-1 in isolated mitochondria by Western blotting. (n=3).*p<0.05 vs control. **(B) and (C)** High glucose and palmitate regulating mitochondrial localization ofDrp-1**(B)** and Mfn 2 **(C)** by immunofluorescence microscopy using anti-Drp-1 and anti-Mfn 2 (green) and Mitotracker (red) and DAPI (blue). Magnified images were shown in insets.

**Figure 3. NaHS and siRNA Drp-1 regulating HPASMCs proliferation rate and phenotype.** (A).NaHS and siRNA Drp-1 induced reduction in HPASMCs proliferation rate. (B). The expression of Collagen I,III and MMP2,9 was decreased with the treatment of NaHS and siRNA Drp-1. *p<0.05 vs control, #p<0.05 vs HG and Pal, and ##p<0.01 vs HG and Pal.

**Fig.1**


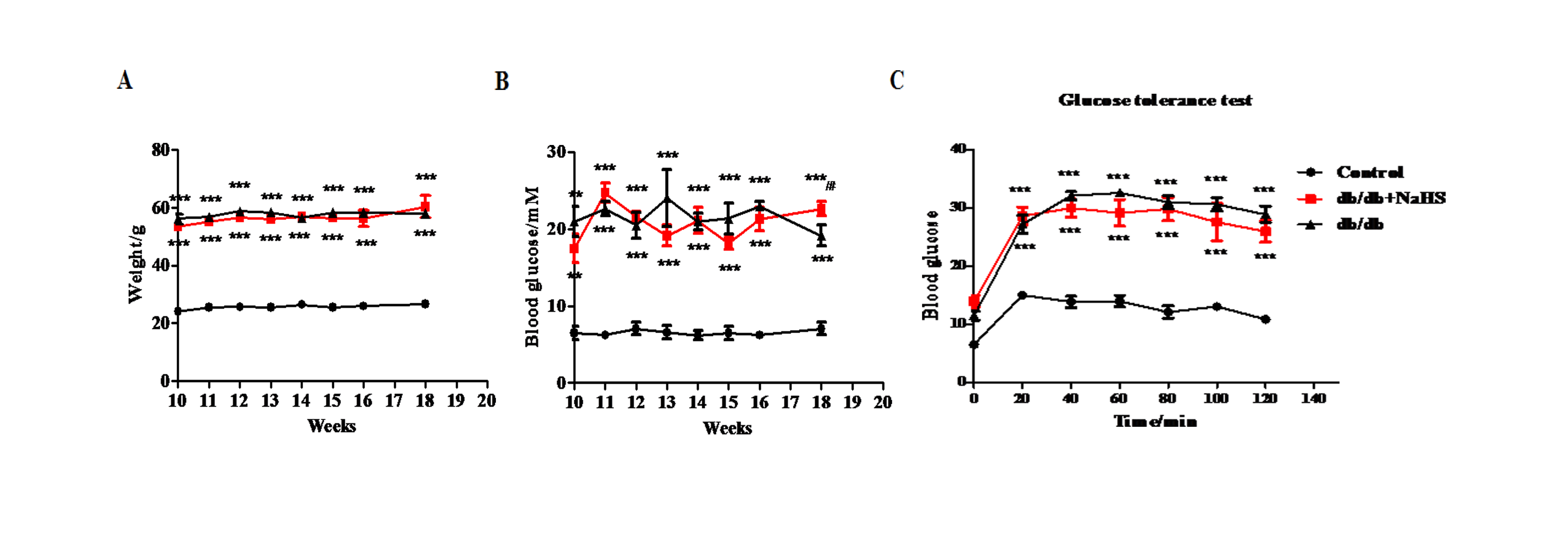


**Fig.2**


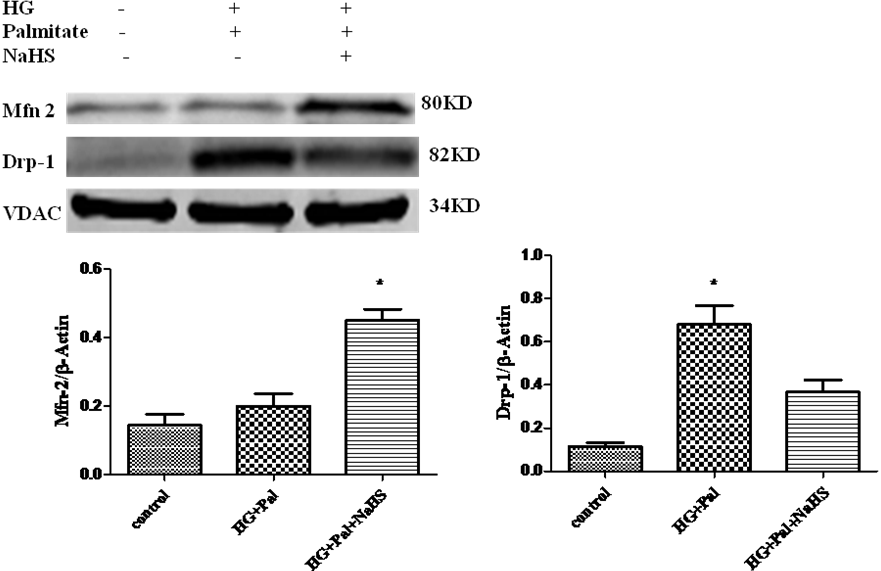


**A**


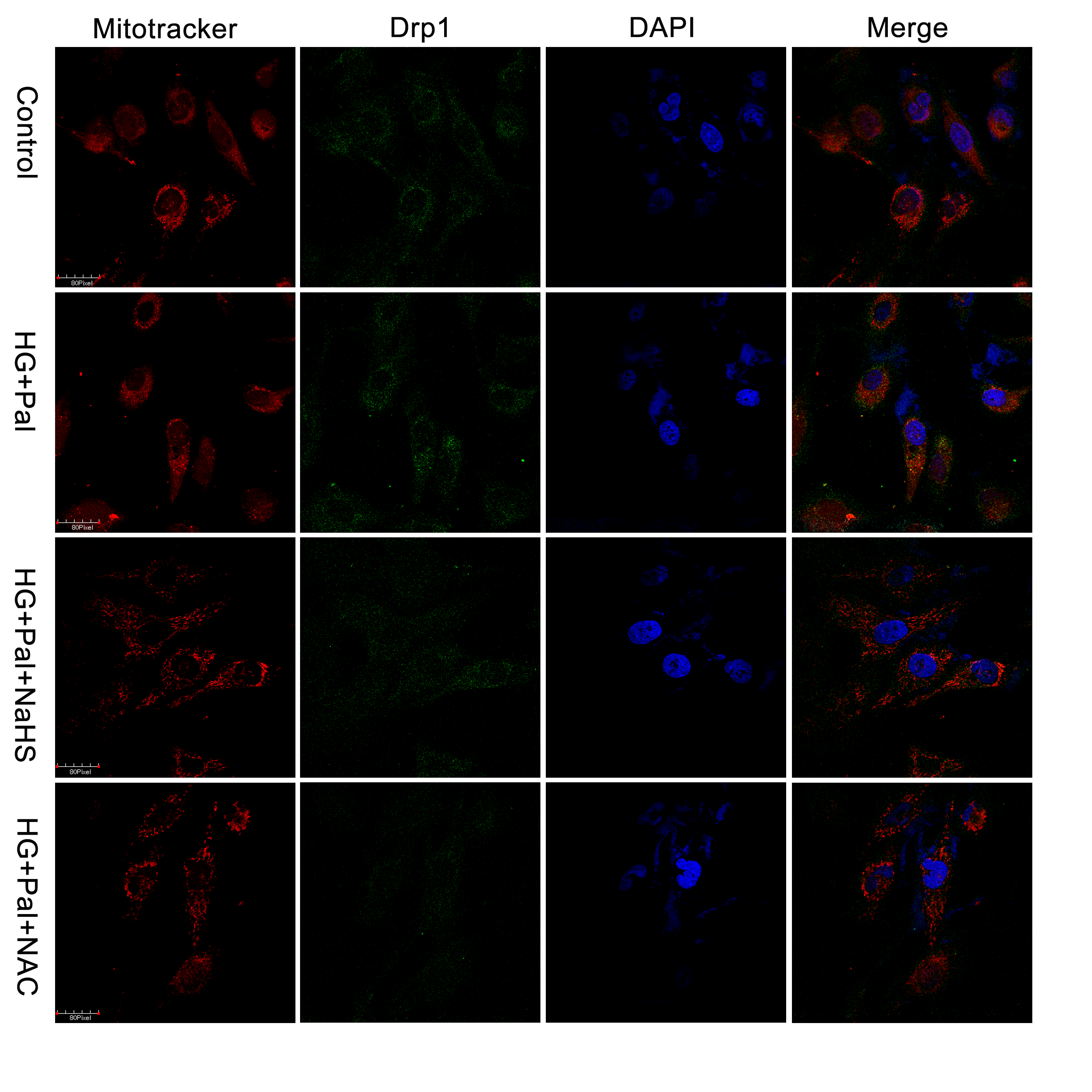


**B**

**C**


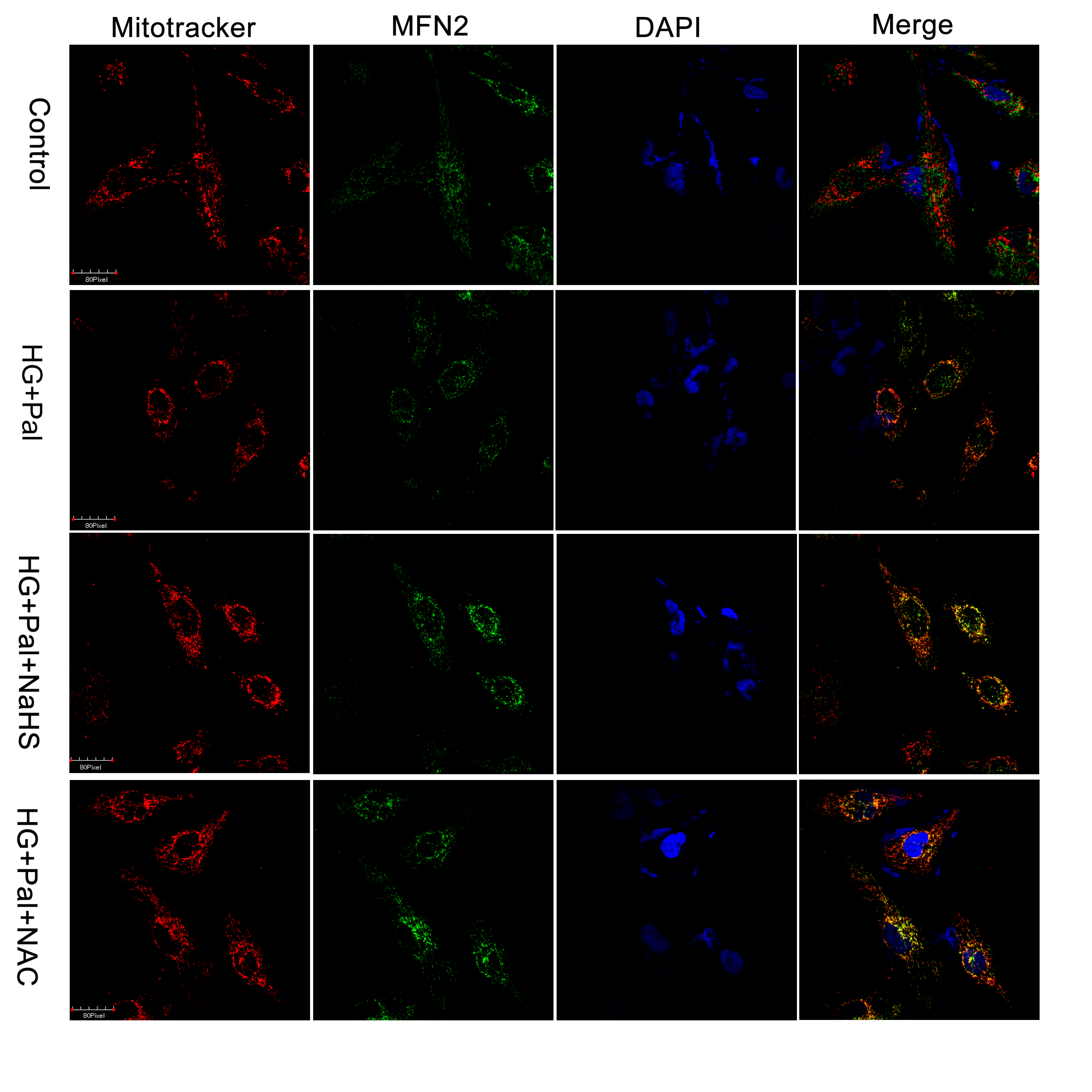


**Fig. 3**


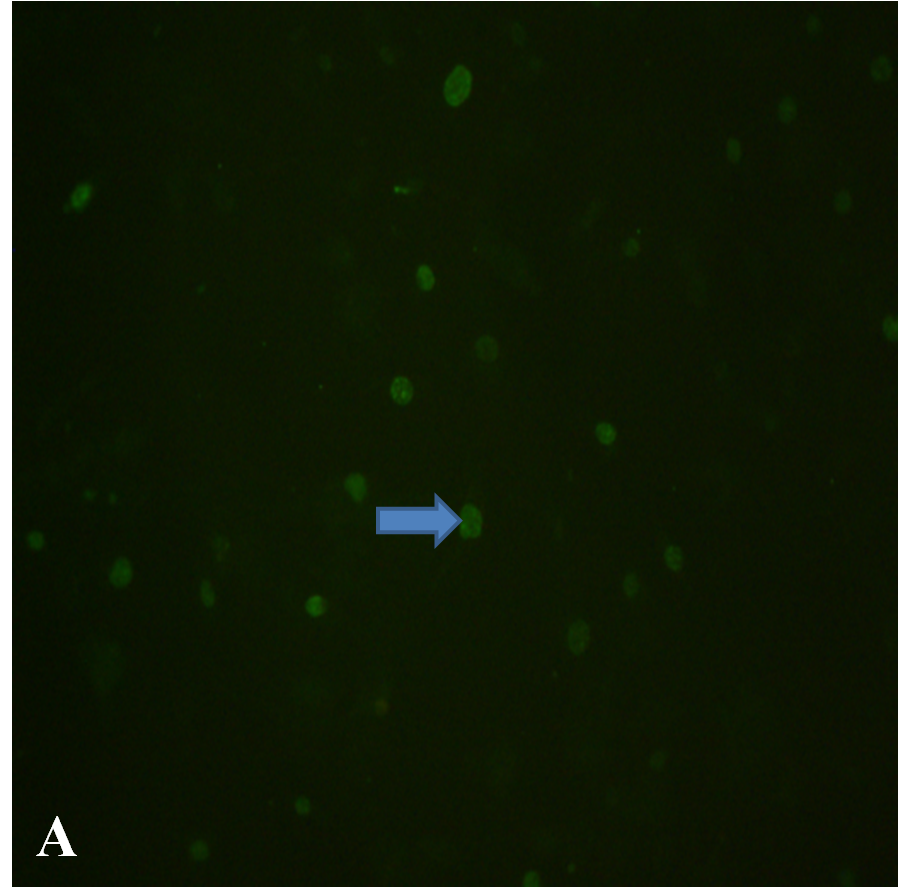

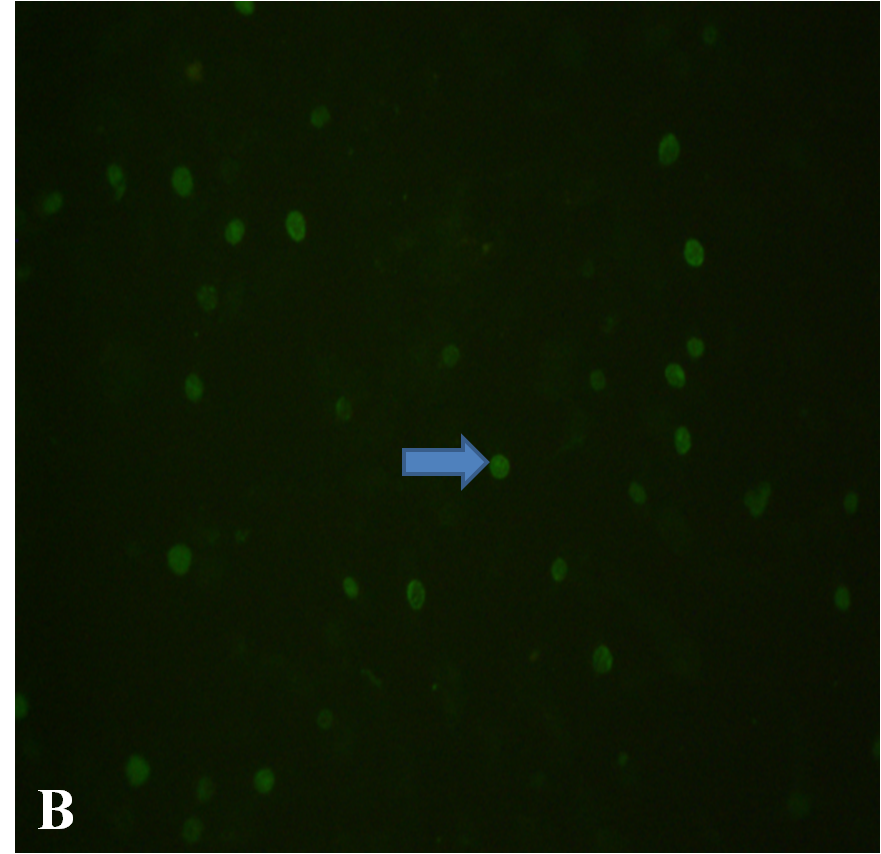

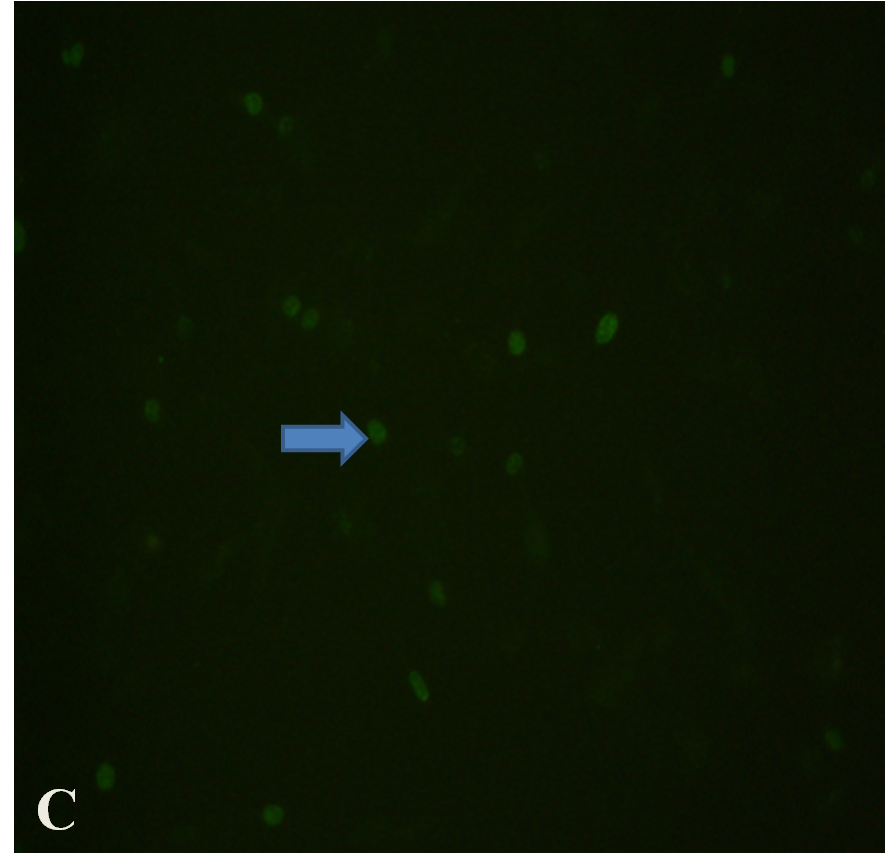

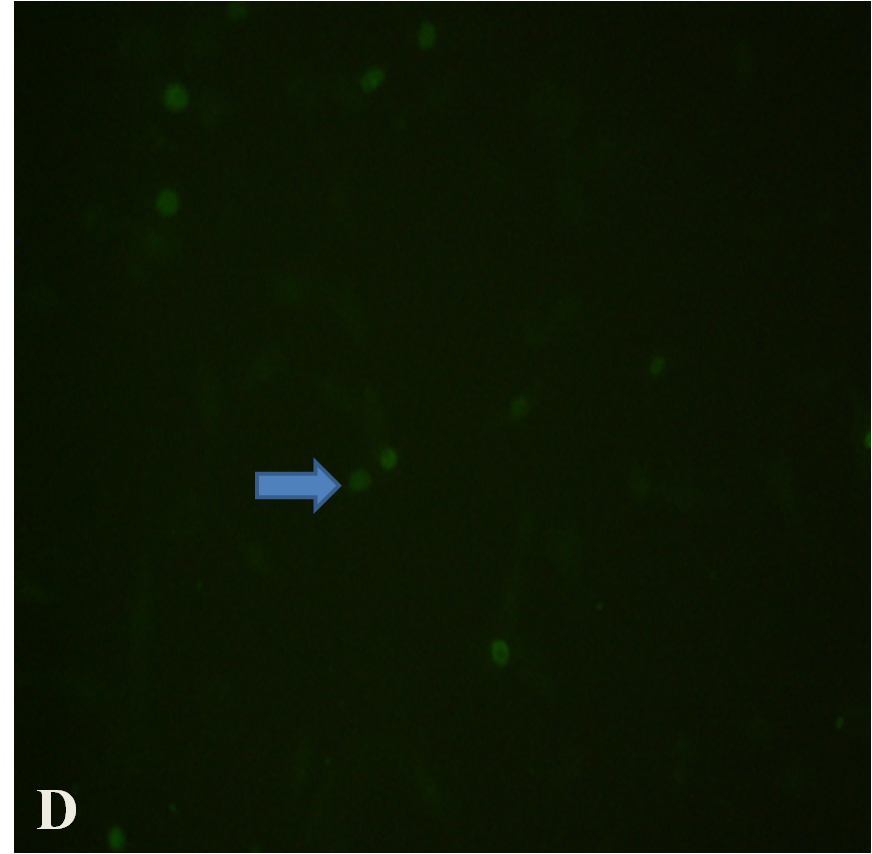

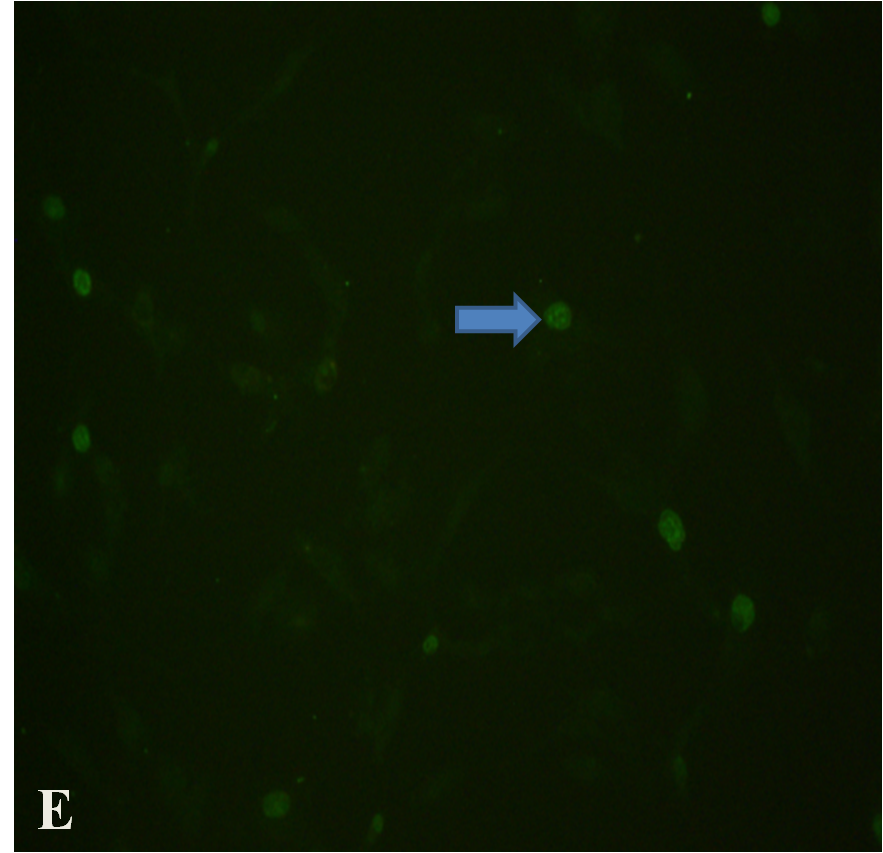


**A**


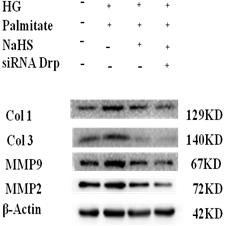


**B**


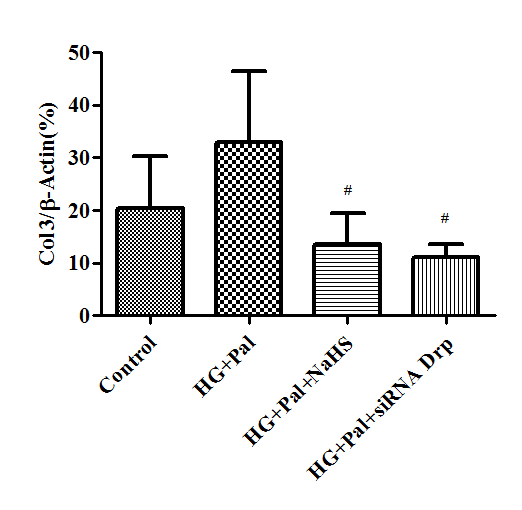


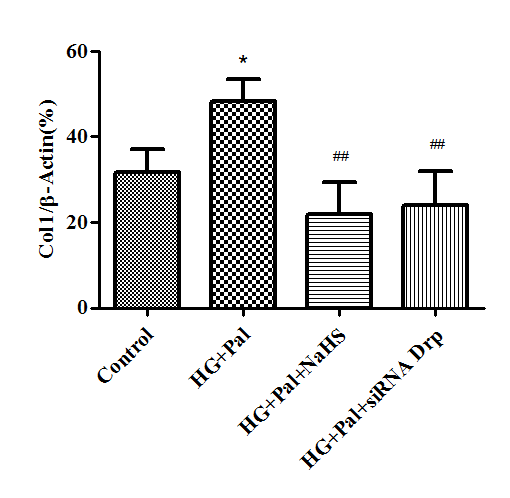


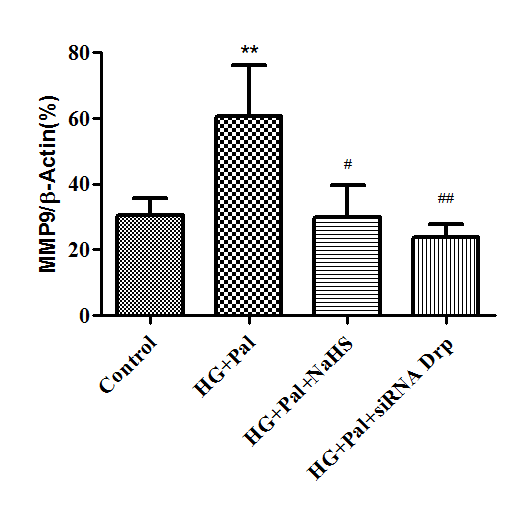


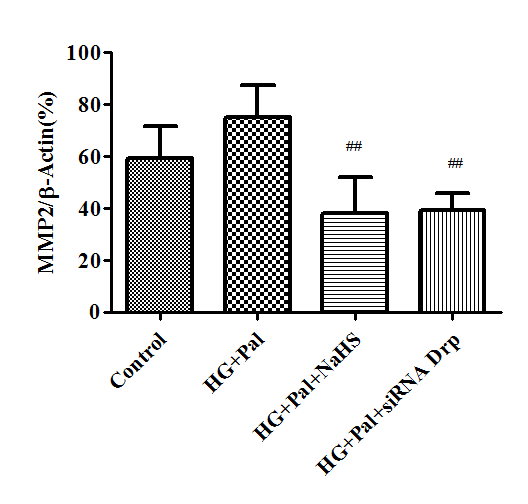

Supplement: Supplementary file 1 — 10.1186/s13578-016-0102-x The effect of exogenous H2S on glucose homeostasis in db/db mice. Figure S2. The expression and localization of Mfn 2 and Drp-1 in mitochondria of HPASMCs treated with high glucose and palmitate. Figure S3. NaHS and siRNA Drp-1 regulating HPASMCs proliferation rate and phenotype. [file 13578_2016_102_MOESM1_ESM.doc]
